# Establishing procedure-specific minimal clinically important difference and patient acceptable symptom state thresholds after anterior combined latissimus dorsi and teres major tendon transfer for irreparable anterosuperior cuff tears: minimum 5-year outcomes

**DOI:** 10.1016/j.jseint.2026.101635

**Published:** 2026-01-27

**Authors:** Chang Hee Baek, Jung Gon Kim, Bo Taek Kim, Chaemoon Lim, Seung Jin Kim

**Affiliations:** Department of Orthopaedic Surgery, Yeosu Baek Hospital, Yeosu-si, Jeollanam-do, Republic of Korea

**Keywords:** Anterior combined latissimus dorsi and teres major tendon transfer, Irreparable anterosuperior rotator cuff tear, Patient-reported outcome measures, Minimal clinically important difference (MCID), Patient acceptable symptom state (PASS), Shoulder function, 5-year outcomes

## Abstract

**Background:**

To date, no prior study has established procedure-specific minimal clinically important difference (MCID) and patient acceptable symptom state (PASS) thresholds for anterior combined latissimus dorsi and teres major (LDTM) tendon transfer in irreparable anterosuperior rotator cuff tears (IASRCTs). This study aimed to establish these patient-centered benchmarks in a cohort with a minimum 5-year follow-up.

**Methods:**

We retrospectively reviewed 31 patients (33 shoulders) who underwent a single-stage anterior LDTM transfer for IASRCTs and completed a minimum 5-year follow-up. Patient-reported outcome measures (PROMs) included the American Shoulder and Elbow Surgeons (ASES) score, visual analog scale (VAS) for pain, Constant score, and activities of daily living requiring internal rotation (ADLIR) score. The MCID was calculated as one-half of the standard deviation of the change score for each PROMs. PASS thresholds were derived from receiver operating characteristic analysis, using postoperative satisfaction as the external anchor.

**Results:**

At a mean follow-up of 83.0 ± 7.4 months, all PROMs improved significantly (*P <* .001). Distribution-based MCID thresholds were 10.5 (ASES), 0.9 (VAS), 10.5 (Constant), and 8.6 (ADLIR). Corresponding MCID achievement rates were 77.4%, 87.1%, 74.2%, and 87.1%, respectively. Anchor-based PASS thresholds were ASES ≥75, VAS ≤2, Constant ≥60, and ADLIR ≥78; these were achieved by 64.5%, 80.6%, 77.4%, and 71.0% of patients, respectively. Age showed a significant negative correlation with ASES MCID (r_pb = −0.53, *P* = .002) and ADLIR MCID (r_pb = −0.41, *P* = .021). Male sex correlated positively with ASES PASS attainment (φ = 0.46, *P* = .010). No other baseline variables were significantly associated with MCID or PASS (all *P* > .05).

**Conclusion:**

This study is the first to establish clinically meaningful MCID and PASS thresholds for anterior LDTM transfer in patients with IASRCTs at a minimum 5-year follow-up. Most patients achieved substantial improvements that were deemed acceptable by the patients. These procedure-specific benchmarks provide practical targets for clinical assessment and patient counseling and serve as reference values for future outcome research.

Irreparable anterosuperior rotator cuff tears (IASRCTs)—defined by combined subscapularis (SSC) and supraspinatus (SSP) insufficiency—severely compromise forward elevation of the shoulder and internal rotation.^40^ Such defects often cause anterosuperior humeral head migration, which predisposes patients to pseudoparalysis and creates formidable surgical challenges.^14^ Débridement or partial repair can yield transient pain improvement; however, functional restoration is seldom achieved.^10^^,^^23^^,^^39^ Moreover, superior capsular reconstruction is contraindicated when the SSC tendon is irreparable.^8^^,^^31^^,^^32^ In younger patients with preserved glenohumeral cartilage who are not yet candidates for reverse shoulder arthroplasty, joint-preserving options remain extremely limited.^10^

Historically, pectoralis major tendon transfer was used for irreparable SSC tears; however, its biomechanical limitations—most notably a nonanatomic line of pull—limit its efficacy in extensive anterosuperior defects.^13^^,^^18^^,^^20^^,^^33^^,^^35^ Meanwhile, isolated anterior latissimus dorsi transfer may improve internal rotation strength but often fails to depress the humeral head effectively when SSP function is compromised.^17^^,^^24^^,^^26^^,^^34^^,^^41^

The combined anterior latissimus dorsi and teres major transfer (LDTM) leverages two powerful scapulohumeral muscles whose excursions and moment arms closely replicate the native SSC vector in both the transverse and coronal planes.^1^^,^^2^ Biomechanical studies show that adding the teres major enhances internal-rotation torque, augments anterior stability, and lowers joint-reaction forces more effectively than an isolated latissimus-dorsi transfer.^5^^,^^6^ Early clinical series report substantial gains in internal rotation function, toileting ability, and pain relief, with tendon healing rates approaching 90%.^3^^,^^4^

Nevertheless, these published outcomes have been confined to absolute score improvements; whether such changes meet thresholds that patients deem clinically meaningful or acceptable has not yet been examined. Patient-centered metrics—namely the minimal clinically important difference (MCID) and the patient acceptable symptom state (PASS)—address this gap by contextualizing change scores and final status from the patient's perspective.^11^^,^^15^^,^^25^ Although MCID and PASS thresholds have been established for several shoulder procedures, procedure-specific values for the anterior LDTM transfer have yet to be defined.

Accordingly, we retrospectively evaluated a consecutive cohort of patients who underwent combined anterior LDTM transfer for IASRCTs, with a minimum 5-year follow-up. The primary aim was to derive procedure-specific MCID and PASS thresholds for four commonly used patient-reported outcome measures (PROMs). Secondary objectives were to quantify mid- to long-term improvements in pain and function, to determine the proportion of patients who achieved these thresholds, and to explore whether demographic or clinical characteristics influenced MCID or PASS achievement. We hypothesized that anterior LDTM transfer would yield durable, patient-perceived improvement and that most patients would achieve both MCID and PASS thresholds at final follow-up.

## Methods

### Study design and patient selection

We retrospectively reviewed 31 patients (33 shoulders) who underwent a single-stage anterior LDTM tendon transfer between April 2015 and January 2019. Eligibility criteria were irreparable IASRCTs and a minimum 5-year postoperative follow-up. Surgical candidacy was established when the following were met: (1) magnetic resonance imaging (MRI) evidence of Goutallier grade^21^ ≥3 fatty degeneration with intraoperative confirmation of irreparability of both the SSC and SSP tendons; (2) persistent shoulder dysfunction and/or pain unresponsive to nonoperative management; (3) intact or repairable infraspinatus (ISP) and teres minor tendons without advanced muscle atrophy; (4) absence of pseudoparalysis, defined by active forward elevation ≥45° with preserved passive range of motion following local anesthetic injection and no evidence of neuromuscular pathology^9^; (5) no radiographic signs of advanced glenohumeral joint degeneration, with inclusion limited to Hamada grade^22^ II or less, and (6) no neurological pathology or severe preoperative stiffness. Sixty-six patients underwent anterior LDTM transfer during the study period. Exclusion criteria included: isolated irreparable SSC tears without SSP involvement (n = 28); <5 years of clinical or imaging follow-up (n = 6); and death before final evaluation (n = 1). Ultimately, 31 patients who met all eligibility criteria and demonstrated irreparability of both the SSC and SSP tendons were included in the final analysis cohort.

### Surgical technique

The combined anterior LDTM transfer technique employed in this cohort has been previously described in detail, including intraoperative images and schematic representations.^1^, ^2^, ^3^, ^4^ In this report, only the key operative steps are summarized. A single senior surgeon performed every procedure under general anesthesia with an interscalene block. Patients were placed in the beach-chair position. Reparability was assessed arthroscopically; tendons were considered irreparable when they could not be mobilized to the native footprint despite complete release. All concomitant ISP tears incidentally identified intraoperatively, despite appearing intact on preoperative MRI, were repaired if feasible.

A standard deltopectoral approach was used from the coracoid to the inferior border of the pectoralis major. The proximal one-third of the pectoralis major was released to improve exposure. The LD and TM tendons were harvested en bloc and whip-stitched with No. 2 Ethibond (J&J MedTech, New Brunswick, NJ, USA). The LDTM tendon was secured with 4.75-mm SwiveLock (Arthrex, Naples, FL, USA) anchors placed distal to the greater tuberosity and around the bicipital groove, with the arm positioned at 45° abduction and full internal rotation. This lateralized fixation optimizes tension and reduces axillary nerve risk compared to conventional approaches.

Postoperatively, patients were immobilized in an abduction brace with the shoulder in internal rotation for 4 weeks. Early movement of the elbow, wrist, and hand was encouraged. Active-assisted shoulder exercises began after brace removal and progressive strengthening was introduced at 3 months. Strenuous activity and sports were restricted for at least 6 months to protect graft healing.

### Patient-reported outcomes

A cohort of 31 patients prospectively completed four validated PROMs both preoperatively and at a minimum of five years after anterior LDTM transfer. The instruments comprised the American Shoulder and Elbow Surgeons (ASES) score, Constant–Murley score, activities of daily living requiring internal rotation (ADLIR) score, and a visual analog scale (VAS) for pain. ADLIR specifically quantifies functional internal rotation during routine tasks such as hand-behind-back reach and personal hygiene. Clinically important change was assessed using the MCID, calculated for each PROM as one-half the standard deviation of the individual change score (postoperative minus preoperative).^11^^,^^15^ This distribution-based approach has been adopted in prior shoulder research.^7^^,^^28^^,^^29^^,^^30^^,^^38^

PASS was determined at the final visit by the anchor question, “Is the current condition of your shoulder acceptable to you?” A dichotomous yes/no reply defined PASS status.^11^^,^^12^^,^^25^ Receiver operating characteristic (ROC) curves were generated for every PROM, and the Youden index (sensitivity + specificity − 1) identified the optimal PASS cutoff. Only PROMs demonstrating adequate discrimination (area under the curve [AUC] ≥ 0.70) were retained.^12^^,^^29^^,^^37^

### Variables

Candidate predictors for MCID or PASS attainment were age, sex, body mass index (BMI), diabetes mellitus (DM), hypertension (HTN), preoperative acromiohumeral distance (AHD) obtained from standardized anteroposterior plain radiographs, anterior glenohumeral subluxation assessed on the same radiographs, and MRI confirmation of an ISP tendon tear. Age, BMI, and AHD were analyzed as continuous variables, whereas sex, DM, HTN, ISP tear, and anterior subluxation were treated as binary factors.

### Power analysis

Sample-size requirements were estimated in advance with G∗Power (version 3.1).^19^ For the primary endpoint—the paired comparison of preoperative and postoperative ASES scores—a two-sided paired *t*-test (α = 0.05, 1 − β = 0.80) based on an anticipated within-subject effect size of Cohen *d*_z_ = 0.60 indicated that at least 27 patients were needed. For the exploratory correlations between baseline characteristics and binary MCID or PASS outcomes, a two-tailed Fisher z calculation targeting |ρ| = .50 under the same error parameters required 29 patients. The final cohort comprised 31 patients, exceeding both thresholds, so no post-hoc power assessment was undertaken.

### Statistical analysis

All analyses were performed in IBM SPSS Statistics 27 (IBM Corp., Armonk, NY, USA) and R version 4.3.2 (R Foundation for Statistical Computing, Vienna, Austria), using the RStudio IDE (Posit Software, PBC, Boston, MA, USA), with the rstatix and pROC packages. Normality of continuous data was evaluated using the Shapiro–Wilk test. Variables meeting normality assumptions are reported as mean ± standard deviation and were compared with either paired- or independent-samples *t*-tests. Non-normal variables were analyzed with the Wilcoxon signed-rank test (paired data) or Mann–Whitney *U* test (independent data). Categorical data are presented as counts (percentages) and were compared with Pearson χ^2^ tests, or Fisher exact tests when expected cell frequencies were <5.

Associations between continuous predictors (age, BMI, AHD) and the binary outcomes of MCID or PASS attainment were assessed using point-biserial correlation (r_pb). Relationships involving binary predictors (sex, DM, HTN, ISP tear, anterior subluxation) were assessed with χ^2^ contingency analyses; φ coefficients were reported as effect sizes.

ROC analysis was used to derive anchor-based PASS cut-offs and, for completeness, anchor-based MCID thresholds. AUCs and 95% confidence intervals were estimated with a bias-corrected percentile bootstrap (2,000 resamples), and DeLong's variance estimate provided an internal check of AUC precision.^16^

## Results

Thirty-one patients (33 shoulders) were followed for 83.0 ± 7.4 months (range, 66-97 months), and complete clinical and patient-reported outcome data were available at final assessment. The mean age at surgery was 66.7 ± 8.3 years, and 41.9% of the cohort was female. Comorbidities comprised DM (19.4%), HTN (51.6%), and a history of smoking (9.7%) (Table I).

**Table I:** Baseline demographic and clinical characteristics.

Variable	Value
Age (yr), mean ± SD (range)	66.7 ± 8.3 (56-82)
Female, n (%)	13 (41.9%)
BMI (kg/m^2^), mean ± SD	24.2 ± 2.3
Arm dominance (dominant arm surgery), n (%)	23 (74.2%)
DM, n (%)	6 (19.4%)
HTN, n (%)	16 (51.6%)
Smoking, n (%)	3 (9.7%)
Preoperative AHD (mm), mean ± SD	8.5 ± 1.7
Preoperative Hamada grade, mean ± SD	1.3 ± 0.5
Preoperative anterior glenohumeral subluxation, n (%)	15 (48.4)
Concomitant ISP tear, n (%)	7 (22.6%)
Follow-up period (month) mean ± SD (range)	83.0 ± 7.4 (66-97)

*SD*, standard deviation; *BMI*, body mass index; *DM*, diabetes mellitus; *HTN*, hypertension; *AHD*, acromiohumeral distance; *ISP*, infraspinatus.

Continuous variables are presented as mean ± SD; categorical data as n/N (%).

By final follow-up, all outcome measures improved significantly relative to baseline (*P <* .001 for each comparison). Mean VAS pain score decreased by 2.7 points, from 4.7 ± 1.4 preoperatively to 2.0 ± 1.2 at final follow-up. The ASES score rose from 47.4 ± 12.3 to 76.3 ± 15.0 (increase, 28.9 points), whereas the Constant score improved from 47.7 ± 9.3 to 66.0 ± 14.3 (increase, 18.3 points). ADLIR score rose from 47.0 ± 12.7 to 74.0 ± 15.4 at final follow-up (increase, 27.0 points). Nineteen of 31 patients (61%) responded **‘Yes’** on the PASS anchor, indicating they felt their postoperative shoulder status was acceptable.

Distribution-based MCID thresholds, defined as one-half of the standard deviation of the score change, were 0.9 for VAS, 10.5 for ASES, 10.5 for Constant, and 8.6 for ADLIR (Table II). MCID achievement rates were 87.1% (VAS), 77.4% (ASES), 74.2% (Constant), and 87.1% (ADLIR).

**Table II:** Distribution-based minimal clinically important difference values and anchor-based patient acceptable symptom state thresholds at 5-year follow-up.

Outcome measure	MCID (½ SD)	% achieved (MCID)	PASS threshold	AUC	95% CI	% achieved (PASS)
VAS	0.9	87.1	2	0.79	0.62-0.93	80.6
ASES	10.5	77.4	75	0.90	0.75-1.00	64.5
Constant	10.5	74.2	60	0.71	0.49-0.89	77.4
ADLIR	8.6	87.1	78	0.86	0.71-0.97	71.0

*MCID*, minimal clinically important difference; *CI*, confidence interval; *PASS*, patient acceptable symptom state; *VAS*, visual analog scale; *ASES*, American Shoulder and Elbow Surgeons score; *ADLIR*, activities of daily living requiring internal rotation; *AUC*, area under the curve; ROC, receiver operating characteristic.

MCID was defined as one-half of the standard deviation of the score change. PASS thresholds were derived from ROC curves (Youden index (sensitivity + specificity − 1)).

**Figure 1:**
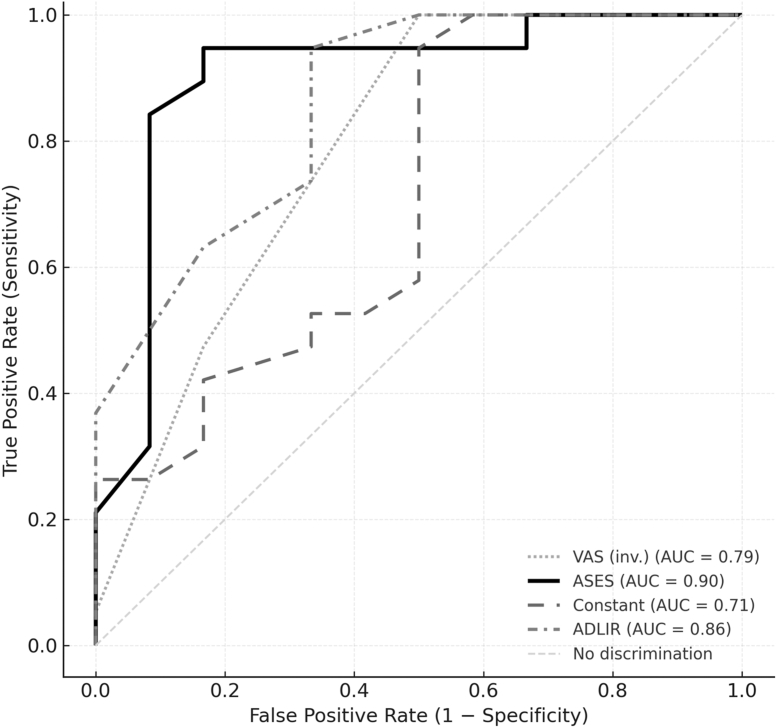
ROC curves identifying PASS thresholds for each PROM. Receiver operating characteristic curves (95% confidence bands shaded) show discrimination of VAS, ASES, Constant, and ADLIR. AUCs: ASES = 0.90, ADLIR = 0.86, VAS = 0.79, Constant = 0.71. Optimal cut-offs were chosen by Youden's index. *ROC*, receiver operating characteristic; *PASS*, patient acceptable symptom state; *PROM*, patient-reported outcome measure; *VAS*, visual analog scale; *ASES*, American Shoulder and Elbow Surgeons score; *ADLIR*, activities of daily living requiring internal rotation; *AUC*, area under the curve.

**Figure 2:**
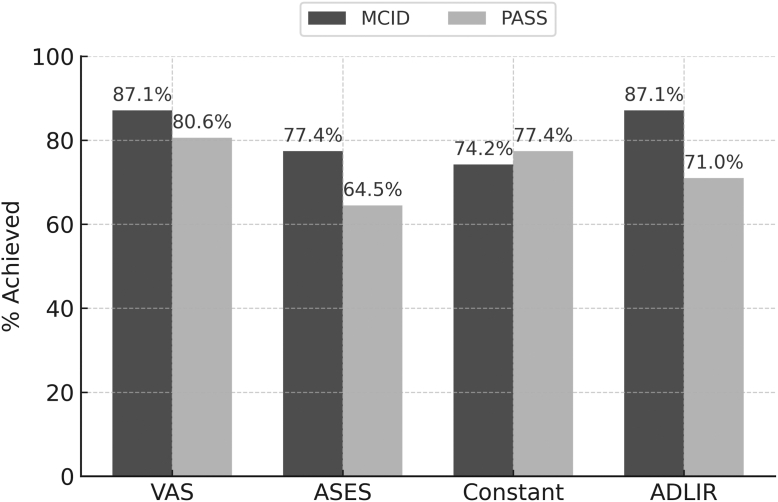
Proportion of patients achieving MCID and PASS for each PROM. A bar graph shows the proportion of patients achieving the MCID (*lighter-colored bars*) and PASS (*darker-colored bars*) thresholds for each PROM (VAS, ASES, Constant, and ADLIR).MCID achievement rates consistently exceeded PASS rates across all PROMs. *MCID*, minimal clinically important difference; *PASS*, patient acceptable symptom state; *PROM*, patient-reported outcome measure; *VAS*, visual analog scale; *ASES*, American Shoulder and Elbow Surgeons score; *ADLIR*, activities of daily living requiring internal rotation.

ROC analysis identified PASS thresholds of VAS ≤2, ASES ≥75, Constant ≥60, and ADLIR ≥78 with corresponding AUC values of 0.79, 0.90, 0.71, and 0.86, respectively (Fig. 1). Notably, the Constant score threshold showed only modest discrimination (AUC 0.71, 95% confidence interval 0.49-0.89), suggesting this cut-off may be less reliable. PASS achievement rates were 80.6% (VAS), 64.5% (ASES), 77.4% (Constant), and 71.0% (ADLIR). Seventeen patients (54.8%) met the PASS threshold for all four PROMs. Furthermore, every patient who deemed their shoulder outcome acceptable (PASS anchor = yes) had met at least three of these PASS benchmarks. (Table II; Fig. 2).

Correlation analysis (Table III) demonstrated that, apart from age and sex, no baseline factor was significantly associated with MCID or PASS attainment. Age was negatively correlated with ASES MCID (r_pb = −0.53, *P* = .002) and ADLIR MCID (r_pb = −0.41, *P* = .021), indicating younger patients were more likely to achieve meaningful improvement in those scores. In contrast, age was not significantly related to PASS status for any outcome (all *P* > .05). Male sex correlated positively with ASES PASS attainment (φ = 0.46, *P* = .010), but no other demographic or anatomic factors – including BMI, DM, HTN, smoking history, preoperative AHD, ISP tear, or anterior subluxation – showed a significant relationship with achieving MCID or PASS for any outcome (all *P* > .05).

**Table III:** Associations between baseline factors and minimal clinically important difference/patient acceptable symptom state attainment.

Variable	VAS MCID	ASES MCID	Constant MCID	ADLIR MCID	VAS PASS	ASES PASS	Constant PASS	ADLIR PASS
Age (yr)	−0.260 (0.158)	**−0.527∗ (0.002)**	−0.201 (0.279)	**−0.412∗ (0.021)**	−0.306 (0.094)	−0.322 (0.077)	−0.245 (0.184)	−0.248 (0.179)
Sex (M/F)	−0.223 (0.214)	0.166 (0.354)	0.096 (0.592)	−0.132 (0.462)	0.080 (0.656)	**0.463∗ (0.010)**	0.010 (0.955)	0.177 (0.326)
BMI (kg/m^2^)	0.126 (0.498)	0.001 (0.996)	0.237 (0.200)	0.058 (0.758)	0.086 (0.644)	0.020 (0.914)	0.187 (0.314)	0.211 (0.255)
DM	0.129 (0.474)	−0.126 (0.483)	−0.271 (0.132)	−0.055 (0.759)	−0.173 (0.335)	−0.149 (0.408)	−0.321 (0.074)	−0.046 (0.796)
HTN	0.271 (0.131)	−0.060 (0.739)	0.019 (0.916)	−0.180 (0.316)	−0.148 (0.411)	−0.178 (0.320)	−0.060 (0.739)	−0.050 (0.779)
Preoperative AHD (mm)	0.114 (0.541)	−0.023 (0.903)	−0.131 (0.482)	0.086 (0.647)	0.024 (0.897)	0.080 (0.669)	−0.114 (0.540)	−0.105 (0.572)
Preoperative ISP tear	0.142 (0.430)	−0.077 (0.667)	−0.034 (0.849)	−0.252 (0.160)	−0.321 (0.074)	−0.244 (0.173)	−0.077 (0.667)	−0.164 (0.360)
Anterior subluxation	0.254 (0.157)	0.060 (0.739)	−0.167 (0.354)	0.180 (0.316)	0.148 (0.411)	−0.091 (0.611)	−0.095 (0.598)	−0.092 (0.609)

*MCID*, minimal clinically important difference; *PASS*, patient acceptable symptom state; *BMI*, body mass index; *AHD*, acromiohumeral distance; *ISP*, infraspinatus; *VAS*, visual analog scale; *ASES*, American Shoulder and Elbow Surgeons score; *ADLIR*, activities of daily living requiring internal rotation; *DM*, diabetes mellitus.

*P* < .05 indicated by an asterisk. Continuous predictors analyzed with point-biserial correlation (r_pb); binary predictors analyzed with φ-coefficient from Fisher's exact or χ^2^ test.

Complications included musculotendinous junction retear of the transferred LDTM in 4 patients (12.9%). Three of these were managed conservatively with scarred healing, whereas 1 patient required conversion to reverse shoulder arthroplasty for persistent symptoms. Superficial infections were noted in two patients (6.5%), and these were treated successfully with surgical débridement and intravenous antibiotics. One patient (3.2%) developed transient axillary nerve palsy, which resolved completely with conservative management.

## Discussion

This investigation is the first mid- to long-term cohort study to establish procedure-specific MCID and PASS thresholds after anterior LDTM transfer for IASRCTs. Previous LDTM series uniformly documented absolute improvements in patient-reported outcomes; however, while some studies reported MCID achievement rates using thresholds established for other rotator cuff procedures, none have derived MCID or PASS values specific to the anterior LDTM transfer. Accordingly, our findings confirm that the LDTM transfer provides sustained improvements in pain and function. Moreover, this study is the first to establish procedure-specific MCID and PASS benchmarks for the anterior LDTM transfer, providing data-driven reference values to evaluate treatment efficacy and patient satisfaction.

Using the half-standard-deviation rule, we derived MCID thresholds of 10.5 points (ASES), 0.9 points (VAS), 10.5 points (Constant), and 8.6 points (ADLIR). Attainment rates were correspondingly high—77.4% (ASES), 87.1% (VAS), 74.2% (Constant), and 87.1% (ADLIR)—underscoring the clinical relevance of these cut-offs. Comparable figures have been documented in other shoulder procedures that relied on the same distribution-based approach: arthroscopic cuff repair cohorts achieved an ASES MCID of 10.5 points with ≈87% success^28^; reverse shoulder arthroplasty series reported thresholds near 11-12 points and ≈89% attainment^7^; and primary shoulder arthroplasty studies described MCIDs of 12-17 points with 80%-90% achievement.^38^ A broad review pooling more than thirty investigations placed the ASES MCID range at 6-17 points.^27^ The alignment of our values with these published intervals supports both the validity and external generalizability of the present benchmarks, while the high achievement proportions highlight the meaningful functional gains realized in this challenging patient population.

In our series, anchor-based PASS thresholds of ASES ≥75, VAS ≤2, Constant ≥60, and ADLIR ≥78 were attained by 64.5%, 80.6%, 77.4%, and 71.0% of patients, respectively. These proportions exceed those typically seen after arthroscopic cuff repair (VAS ≤2.5, 68%) and superior capsular reconstruction (ASES ≥70, 60%). Prior shoulder studies that employed the same yes/no satisfaction anchor have reported ASES PASS cut-offs spanning the high-50s to mid-80s, with achievement rates between roughly 49% and 72% at 2-5 years.^7^^,^^29^^,^^36^ Our ASES threshold of 75 (achieved by 64.5% of patients) and the VAS cut-off of ≤2 (80.6% achieved) therefore fell well within the published range. Collectively, these findings indicate that the procedure-specific PASS values identified for anterior LDTM transfer are consistent with external benchmarks and capture the expectations of patients who choose this joint-preserving option over reverse arthroplasty.

These parallels suggest that, regardless of procedure, patients tend to consider ASES scores in the high-60s to low-80s as an acceptable symptom state after major shoulder reconstruction. Our results, which reflect a relatively active and high-demand population, support the external validity and applicability of these PASS benchmarks to the LDTM context. Therefore, both the MCID and PASS values established in this study are not only internally robust but also highly consistent with published benchmarks from related procedures, underscoring their clinical relevance and utility. These validated thresholds offer a reliable framework for outcome assessment and patient counseling following LDTM transfer, and they should facilitate more meaningful interpretation of future clinical results in this domain.

Correlation analysis (Table III) demonstrated that, apart from age and sex, no other demographic or anatomic variables—including BMI, DM, HTN, smoking history, preoperative AHD, concomitant ISP tear, or preoperative anterior subluxation—were significantly associated with attaining an MCID or PASS threshold for any outcome. Age showed a significant negative correlation with ASES MCID (ρ = −0.53, *P* = .002) and ADLIR MCID (ρ = −0.41, *P* = .021), indicating that younger patients were more likely to attain meaningful improvement in those scores. However, age was not significantly associated with PASS for any outcome (all *P* > .05), suggesting that although younger individuals more readily attain relative improvements (MCID), older patients can still reach an acceptable final state (PASS) despite smaller change magnitudes. Likewise, male sex was positively associated with attaining the ASES PASS threshold (φ = 0.46, *P* = .010), indicating that male patients were more likely to attain a satisfactory ASES outcome. These results suggest that, aside from age and sex, most baseline patient characteristics did not independently influence the likelihood of achieving MCID or PASS in this cohort.

This study has several limitations. Our sample size, while among the largest for this procedure with long-term follow-up (n = 31 patients, 33 shoulders), remains modest and limits subgroup analyses. The retrospective design introduces potential selection and recall bias, although all data were collected prospectively at standardized intervals. Because the MCID values were derived using a distribution-based method, they do not necessarily reflect the patient's perceived improvement. We addressed this limitation in part by incorporating an anchor-based PASS analysis, using a simple yes/no patient satisfaction question. This anchor-based method allowed us to derive thresholds directly linked to patients' perception of acceptable shoulder function, thereby enhancing the clinical applicability and patient-centered perspective of our results. Our use of a binary satisfaction anchor follows established practice in shoulder research.^7^^,^^29^^,^^36^^,^^42^ Nonetheless, future investigations might benefit from incorporating more nuanced instruments—such as a Global Rating of Change scale—to capture gradations in patient-reported improvement and further refine the interpretability and external validity of these outcome thresholds. Finally, the absence of a direct comparison group (eg, reverse shoulder arthroplasty or isolated LD transfer) precludes head-to-head efficacy assessment, although our primary aim was to establish reference values specifically for LDTM transfer.

## Conclusion

This study is the first to establish clinically relevant, procedure-specific MCID and PASS thresholds following anterior LDTM tendon transfer for IASRCTs, assessed at a minimum 5-year follow-up. MCID benchmarks were identified as 10.5 points (ASES), 10.5 points (Constant), 0.9 points (VAS), and 8.6 points (ADLIR), with high achievement rates observed. PASS thresholds, reflecting satisfactory clinical outcomes, were established as ASES ≥75, Constant ≥60, VAS ≤2, and ADLIR ≥78. These validated reference values provide practical criteria to interpret postoperative improvements, enhance patient counseling, and facilitate comparative assessments in future clinical research and practice.

## Acknowledgment

The authors extend their gratitude to Sung Hak Choi, Yeong Ran Seo, and Seung Hwan Oh who served as research coordinators and provided valuable assistance in collecting clinical and surgical data.

## Disclaimers

Funding: No funding was disclosed by the authors.

Conflicts of interest: The authors, their immediate families, and any research foundation with which they are affiliated have not received any financial payments or other benefits from any commercial entity related to the subject of this article.
